# Safety and efficacy of a purified canine immunoglobulin G formulation for treatment of 76 cats clinically affected by the Australian paralysis tick (*Ixodes holocyclus*)

**DOI:** 10.1111/avj.13194

**Published:** 2022-07-04

**Authors:** AM Padula

**Affiliations:** ^1^ Padula Serums Pty Ltd Bairnsdale Victoria 3875 Australia; ^2^ Australian Venom Research Unit, Department of Pharmacology and Therapeutics, Faculty of Medicine, Dentistry and Health Science University of Melbourne Parkville Victoria 3010 Australia

**Keywords:** cats, immunoglobulin, *Ixode holocyclus*, tick antiserum, tick paralysis

## Abstract

Acute adverse reactions in cats administered unrefined canine paralysis tick (*Ixodes holocyclus*) antiserum are commonly observed by veterinarians and can lead to significant morbidity and potentially fatal. A purified antiserum canine IgG concentrate was chromatographically prepared and aseptically formulated in single doses containing the equivalent of 5 mL of unrefined tick antiserum (TAS). The IgG was used for slow intravenous infusion into clinically affected cats at multiple veterinary clinics on the eastern seaboard of Australia. Overall, 72/76 (95%) of cats survived hospital discharge, an efficacy comparable to published data. A subset of 22 cats previously treated with unrefined TAS and considered high risk were included in the dataset. The safety profile was excellent with 0/76 acute adverse reactions although 2/76 (2.6%) developed mild facial swelling within 2 h of infusion that responded to the antihistamine. In conclusion, cats intravenously infused with purified IgG from canine TAS did not exhibit the expected frequency of acute adverse reactions during infusion and it was both safe and effective for the treatment of tick paralysis in cats.

**Abbreviations**
IgG: immunoglobulin G
PTAS: purified tick antiserum
TAS: tick antiserum

Cats frequently succumb to lower motor neurone paralysis from salivary gland neurotoxins found within engorged Australian paralysis ticks (*Ixodes holocyclus*).^1^ Alongside supportive hospital care, the core treatment for clinically affected cats is the administration of canine origin tick antiserum (TAS) to neutralise circulating tick neurotoxins.^2^ Despite the apparent benefit of TAS, acute and sometimes fatal adverse reactions occur within minutes of administration and were reported to occur in 161/1735 (9.3%)^3^ and 375/6074 (6.2%)^4^ of cats.

The clinical description of acute TAS reactions in cats appears primarily anaphylactic in nature.^5^ The signs described include rapid onset pawing at the face, gagging, vomiting, salivation, piloerection on the back of the neck, tachycardia, collapse, severe respiratory distress and cardiac arrest. Clinical signs in cats sensitised to bovine serum albumin after intravenous challenge were similar to that of an acute TAS reaction and had a fatal outcome.^6^

The potential for acute adverse reactions during TAS administration to cats is recognised amongst veterinary practitioners and occurs despite premedication with corticosteroids, antihistamines and adrenaline.^7^, ^8^ Cats that experienced a TAS reaction were reported to have a higher risk of mortality overall and risk versus benefit approach was advocated for its use.^3^ Previous treatment with TAS appears to increase the risk of an acute reaction occurring, likely through sensitisation to the foreign protein.^3^ TAS reactions in cats were associated with 5.3 times increased incidence of mortality by day 5 of hospitalisation in the largest study to date in cats.^3^

The problem of acute adverse reactions to antiserum administration is not unique to TAS in cats and a similar problem was noted over 100 years ago during the initial development of serum therapy against diphtheria toxin in humans.^9^ Unrefined horse serum from donor horses immunised against diphtheria toxin was widely used to treat children and although spectacularly successful in some cases, acute reactions and deaths within minutes of administration soon became recognised as a significant problem.^9^ However, a reduction in the frequency of acute reactions to heterologous antiserum administration occurred when crude serum was fractionated into purified immunoglobulin (IgG) formulations consisting of either purified whole IgG or enzymatically digested into F(ab′)_2_ fragments.^10^ Experimental studies in guinea pigs, a species highly sensitive to sensitisation and anaphylaxis, demonstrated that removal of non‐IgG protein by a two‐step purification process almost entirely prevented anaphylaxis following intravenous challenge.^11^ Commercially available TAS in Australia is unrefined crude serum and methods of production have changed little since the first published method of production.^12^

This study was undertaken to test the hypothesis that fractionation of TAS into a highly purified immunoglobulin formulation would reduce the risk of acute adverse reactions when administered intravenously to cats whilst retaining efficacy.

Commercial TAS was purchased and purified using a two‐step process consisting of chemical precipitation^13^ of non‐IgG protein followed by chromatographic adsorption on Diethylaminoethyl Sephadex.^11^ The finished product was extensively diafiltered against 0.01 M phosphate buffered saline pH 7.4 to remove phenol and other low molecular weight substances, concentrated by ultrafiltration, sterile filtered and aseptically dispensed into sterile 10 mL glass vials presented as single doses. Potency testing was performed by indirect ELISA^14^ using a partially purified *I. holocyclus* neurotoxin adsorbed antigen. Endotoxin, pH, sterility, total IgG and purity of the final bulk were assessed by standard pharmacopeial methods. Preservative‐free single‐dose vials were prepared to contain not less than 2,500 units/vial of anti‐*I. holocyclus* IgG in a volume of 5 mL. The total unit was chosen to be equivalent in neutralising capacity to 5 mL of TAS.

Veterinarians were invited to participate in the study of purified tick antiserum (PTAS) in cats, to directly substitute the PTAS‐like‐for‐like with TAS and to follow their usual clinical treatment protocol. A dose recommendation of one vial was made and administration of additional vials was made at the discretion of the attending veterinarian. Clinical severity scores of gait and respiration were performed 1–4 by the clinician.^3^ A detailed record during each PTAS intravenous infusion of heart rate, respiratory rate, and SPO_2_ was made. Acute adverse reactions were defined as those occurring within 15 min of infusion and delayed reactions within 2 h. Veterinarians submitted clinical notes shortly after treating each case, PTAS infusion records and feedback. The study was conducted under the remit of the Australian Pesticides and Veterinary Medicines Authority (APVMA) research permit PER7250.

The PTAS purification process was reproducible. The final IgG formulation migrated on an agarose gel as a single peak in the gamma globulin zone with the complete absence of canine serum albumin (Figure 1). The visual appearance of PTAS in the vial was a colourless, clear and particle‐free liquid.

**Figure 1:**
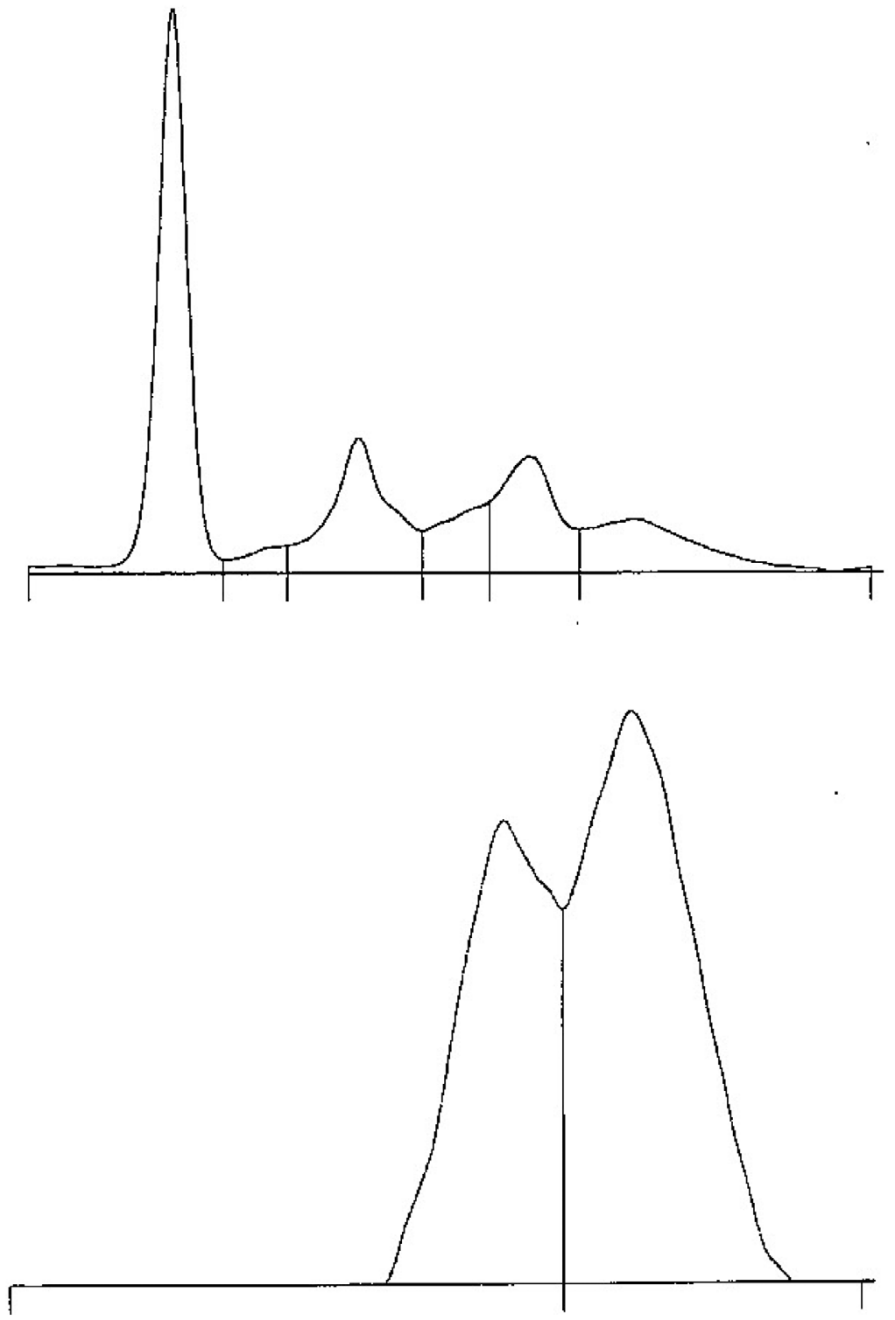
Agarose gel electrophoresis profiles demonstrated complete removal of albumin from TAS. (Top) serum protein migration of unrefined TAS and (bottom) PTAS with the migration of gamma globulin only. Albumin migrates strongly towards the positive electrode and is the first tall peak on the left side of unrefined TAS. PTAS, purified tick antiserum; TAS, tick antiserum.

Survival rates at discharge varied as expected with severity (Table 1) but were unaffected by a dose of PTAS (Table 2). One cat died in hospital and three cats were euthanised on cost and prognosis. No acute adverse reactions were observed during PTAS infusion although two cats did develop delayed facial swelling which responded to antihistamine treatment. A subset of 22 cats treated with PTAS had a prior history of previously receiving TAS on 1–3 occasions and were speculated to be at a higher risk for a reaction, however, no acute reactions were observed.

**Table 1:** Survival to hospital discharge, incidence of acute and delayed reactions in cats receiving purified tick antiserum

Gait score	No. cases (% total)	No. survived to discharge (%)	No. acute reaction (%)	No. delayed reaction (% total)
1	22 (29)	22 (100)	0 (0)	1 (1.3)
2	30 (39)	29 (97)	0 (0)	1 (1.3)
3	19 (25)	18 (95)	0 (0)	0 (0)
4	2 (2.6)	0 (0)	0 (0)	0 (0)
NR	3 (3.9)	3 (100)	0 (0)	0 (0)
Total	76 (100)	72 (95)	0 (0)	2 (2.6)

Percentages rounded up.

NR, not recorded.

**Table 2:** Dose of purified tick antiserum and case outcome

No. vials administered	No. cases (% total)	No. survived to discharge (%)
1	39 (51)	38 (97)
>1	36 (47)	33 (92)
NR	1 (1.3)	1 (100)
Total	76 (100)	72 (95)

The safety profile was excellent with no acute adverse reactions observed in 76 cats treated with 1 to 2 vials. Veterinary practice protocols varied in the duration of infusion and ranged between 30 to 240 min. Facial swelling, presumably caused by angioedema, is a recognised issue with homologous and heterologous plasma transfusion in cats and dogs.^15^ An overall survival rate of 97%^3^ was reported for 2077 cats treated for tick paralysis which is similar to the 95% observed here for PTAS. Administration of TAS to cats was reported to increase survival rates although cats that experienced an acute adverse reaction were more likely to die.^3^ Cats readily make IgG to canine albumin (Padula, unpublished data) contained in TAS. It is likely that IgE specific to canine albumin is also generated and sensitises cats to subsequent anaphylaxis; although fatal acute reactions have been reported in cats that have never been treated with TAS.^3^

Cats previously treated with unrefined TAS should based on theory, be at higher risk of acute anaphylactic reactions to subsequent TAS infusions. Although the dataset presented here is small, cats previously treated with TAS experienced no acute adverse reactions to PTAS. This observation is encouraging and supports the use of PTAS in this potentially higher‐risk group has to merit in reducing infusion risk. However, there may be benefits other than explicitly in cats that have had prior TAS treatment because prior exposure does not explain those adverse reactions reported in naïve cats. Further studies to examine specific IgE responses and their role in acute reactions are required.

A number of APVMA licensed highly effective tick paralysis preventatives have become available since 2018 to cat owners and veterinarians, focusing the clinical need on refinement of ever more effective and safer treatments for those feline cases that do present to veterinarians.^2^

This study has provided preliminary evidence that the use of PTAS in cats can prevent acute adverse reactions from occurring compared to rates in published data, without compromising efficacy.

## Conflicts of interest and sources of funding

PTAS was manufactured by Padula Serums, a company owned by the author Andrew Padula. Boehringer Australia generously provided funding to undertake this research with the endorsement of the Australian Paralysis Tick Advisory Panel.
